# Factors influencing the frequency, knowledge, attitudes and practices of antibiotic use in commercial layer chicken farms, Tanzania

**DOI:** 10.3389/frabi.2025.1571096

**Published:** 2025-04-28

**Authors:** Siha Mdemu, Augustine B. Matondo, Jens Peter Christensen, Ahmed E. Amasha, Helena A. Ngowi, Erica Westwood, Sunday O. Ochai, Hezron E. Nonga, Kristina Osbjer, Robinson H. Mdegela

**Affiliations:** ^1^ Department of Veterinary Medicine and Public Health, College of Veterinary Medicine and Biomedical Sciences, Sokoine University of Agriculture, Morogoro, Tanzania; ^2^ Tanzania Veterinary Laboratory Agency, Vaccine Institute, Kibaha, Pwani, Tanzania; ^3^ Department of Veterinary Anatomy and Pathology, College of Veterinary Medicine and Biomedical Sciences, Sokoine University of Agriculture, Morogoro, Tanzania; ^4^ Department of Veterinary and Animal Sciences, Faculty of Health and Medical Science, University of Copenhagen, Frederiksberg, Denmark; ^5^ University Farm, Sokoine University of Agriculture, Morogoro, Tanzania; ^6^ International Centre for Antimicrobial Resistance Solutions (ICARS), Copenhagen, Denmark; ^7^ Department of Veterinary Tropical Diseases, Faculty of Veterinary Science, University of Pretoria, Onderstepoort, South Africa; ^8^ Antimicrobial Research Unit, College of Health Sciences, University of KwaZulu-Natal, Durban, South Africa

**Keywords:** KAP, AMR, layer farms, cross-sectional, Tanzania

## Abstract

**Introduction:**

Indiscriminate use of veterinary antibiotics significantly contributes to the current antibiotic resistance in the world. The primary objective of this study was to explore the factors that could influence knowledge, attitudes and practices of antibiotic use in commercial layer farms.

**Methods:**

A cross-sectional study was conducted to evaluate antibiotic use patterns and their associated factors among layer chicken farmers in Tanzania. The study surveyed 205 farmers randomly selected from three regions: Unguja, Morogoro, and Dar es Salaam. Data were analysed using descriptive statistics, while negative binomial and multiple linear regression models were employed to identify factors influencing antibiotic usage patterns.

**Results:**

A widespread use of antibiotics was revealed, with 97.1% of farmers using antibiotics for treatment, prophylaxis and/or increasing egg productivity. The most commonly used antibiotics were oxytetracycline (63%), doxycline-tylosin combination (29.8%) and enrofloxacin (22.4%). Notably, 95.6% of farmers reported that they do not observe withdrawal periods. Assessment of farmers' knowledge, attitudes, and practices yielded mean scores of 55.5%, 69.1% and 50.9% respectively. Furthermore, older adults and individuals with primary education were more likely to have higher attitude scores. Geographic location and flock size are among other factors that are likely to influence knowledge and attitudes towards antibiotic use. Higher frequency of antibiotic use was significantly associated with young adults, medium-scale farm operators, and farmers in Morogoro region (compared to the other two regions).

**Conclusion:**

The frequency, knowledge, attitude and practices related to the use of antibiotics were affected by scale of production, location, age, and education. These findings provide insights into antibiotic stewardship among layer farmers that could suggest future multifaceted interventions to promote prudent use of antibiotics, hence mitigating risk of antibiotic resistance.

## 1 Introduction

Commercial layer chickens are major source of eggs and income for the industry stakeholders (Khan et al., 2022). In Tanzania, commercial layer chicken production is a rapidly growing industry owing to the increasing demand for eggs as a versatile and affordable source of protein (Wilson et al., 2022). The number of eggs produced in the country has increased at an annual average rate of 12.4% from 3.2 billion in 2018 to 6.4 billion in 2024 (Ndaki, 2021). However, intensive commercial layer production in resource-limited countries like Tanzania is faced by challenges like climate stress, poor productivity, diseases, unreliable quality feed availability and limited extension services (Nonga et al., 2009). To overcome these challenges, farming practices may result in misuse or overuse of antibiotics that are readily available in veterinary shops (Komba, 2024; Yakubu et al., 2024). Annually, Tanzania uses more than 1.2 million kilograms of antibiotics to support poultry production (Sangeda et al., 2021). Studies in Tanzania have reported a high prevalence of antibiotic residues and the residue concentrations in eggs ranges between 0.103 – 1655 µ/g (Nonga et al., 2009; Mubito et al., 2014a; Mosha, 2017).

It is predicted that by 2030, antimicrobial use in livestock will rise by 8% and 25% in the globe and Africa respectively (Pokharel et al., 2020). The use of antibiotics in animals remain higher in Africa and other developing countries including Tanzania because, unlike in humans, antibiotics are frequently used in animals not only for treatment purpose but also for prophylaxis and to increase production (Mubito et al., 2014b; Pokharel et al., 2020).

Antimicrobial resistance (AMR) is a global issue threatening both human and animal health as well as the productivity of livestock (Magnusson, 2021). Evidence supports the spread of microorganisms resistant to antibiotics from animals to humans or vice versa through direct contact or environment (Lazarus et al., 2015; Pirolo et al., 2019; Magnusson, 2021). It is estimated that the global human deaths associated with AMR will increase from 4.71 million in 2021 to 8.22 million in 2050 (Naghavi et al., 2024). Although AMR challenges are evident worldwide, the threat is of particular concern in the developing countries, notably Africa, where the burden remains the highest (Sartorius et al., 2024). In Tanzania, studies reveal that AMR is a problem across animals, humans and the environment (Moremi et al., 2016; Katakweba et al., 2018; Kissinga et al., 2018). High levels of resistance to the majority of the commonly used veterinary antibiotics including tetracyclines, sulphonamides, penicillins and fluoroquinolones has been frequently reported in the country suggesting a wide use of these antibiotics in the country (Rugumisa et al., 2016; Kiiti et al., 2021; Maganga et al., 2022).

Despite the extensive use of antibiotics, there is inadequate information on the treatment regimens and the determinants of antibiotic use in commercial layer chickens in Tanzania. This study investigated antibiotic usage patterns in commercial layer farms across selected districts, examining factors influencing farmers’ knowledge, attitudes, and practices regarding antibiotic use. The research also analysed variables affecting treatment frequency to identify potential intervention strategies for reducing antibiotic usage, thereby addressing AMR concerns while maintaining layer productivity. These insights aim to inform evidence-based interventions and policy development for AMR mitigation in commercial layer operations.

## 2 Materials and methods

### 2.1 Study areas

This study was carried out in Dar es Salaam, Morogoro and Unguja urban and peri-urban areas of Tanzania from March to June 2023. Dar es Salaam is the region with the largest population in the country covering an area of 1,393 km^2^ located in the eastern Indian Ocean coast of Africa with a population of 5,383,728 inhabitants at a density of 3,865 per km^2^ according to the 2022 Tanzania population census (NBS, 2022). The region is characterized by tropical climatic conditions, with hot and humid weather throughout the year. It has a heavy rainy season (from March to May), a short rainy season (from November to January) and a dry season (from June to October). The annual average rainfall and temperature is 1150 mm and 32°C respectively. Morogoro Municipality has a total area of 540 km^2^ with a population of 471,409 people at a density of 873 per km^2^. It is located at an elevation of 1670 feet above sea level receiving an annual rainfall of 269 mm and temperature of 25°C. Unguja is the largest and most populated island of the Zanzibar located at a latitude of 5°72′ - 6°48′ south and a longitude of 39°30′ - 39°51′ east in the Indian ocean with an area of about 1,666 km^2^ and human population of 1,346,332. The island is characterised by a tropical monsoon climate with two main seasons of rainfall (March to May and November to January) while the months in between receiving less rain. The three localities were chosen due to their ecological characteristics that create a favourable environment for small-scale to large-scale commercial layer farming.

### 2.2 Study design and population

A cross-sectional survey was carried out from March to June 2023. The study included primary decision-makers responsible for flock management and treatment protocols from farms with more than 200 layer chickens. Selection criteria required participants to be over 18 years and willing to share farm information.

### 2.3 Sample size

The sample was estimated using the formula for finite population (Krejcie and Morgan, 1970). Based on registers from the District Veterinary Offices the number of commercial layer chicken farms was estimated at 395 (282 in Dar es Salaam, 64 in Morogoro and 38 in Unguja). The prevalence of inappropriate use of antibiotics was estimated as 50% based on previous studies (Azabo et al., 2022). Assuming an acceptable error of 5% at a 95% confidence level, the resulting minimum sample size for Dar es Salaam, Morogoro and Unguja was 142, 32 and 14 respectively.

### 2.4 Data collection tool and procedure

A semi-structured questionnaire was used to collect data at the farm level. The questionnaire was divided into sections consisting of farm characteristics, socio-demographic characteristics of the interviewee, knowledge about antibiotic use, practices related to antibiotic use and attitude towards antibiotic use in commercial layers. The questionnaire was pre-tested in a group similar to the anticipated respondents for validity, question framing, relevance, and sequencing and was corrected accordingly. The questionnaire was administered through face-to-face interviews using Swahili language.

**Figure 1:**
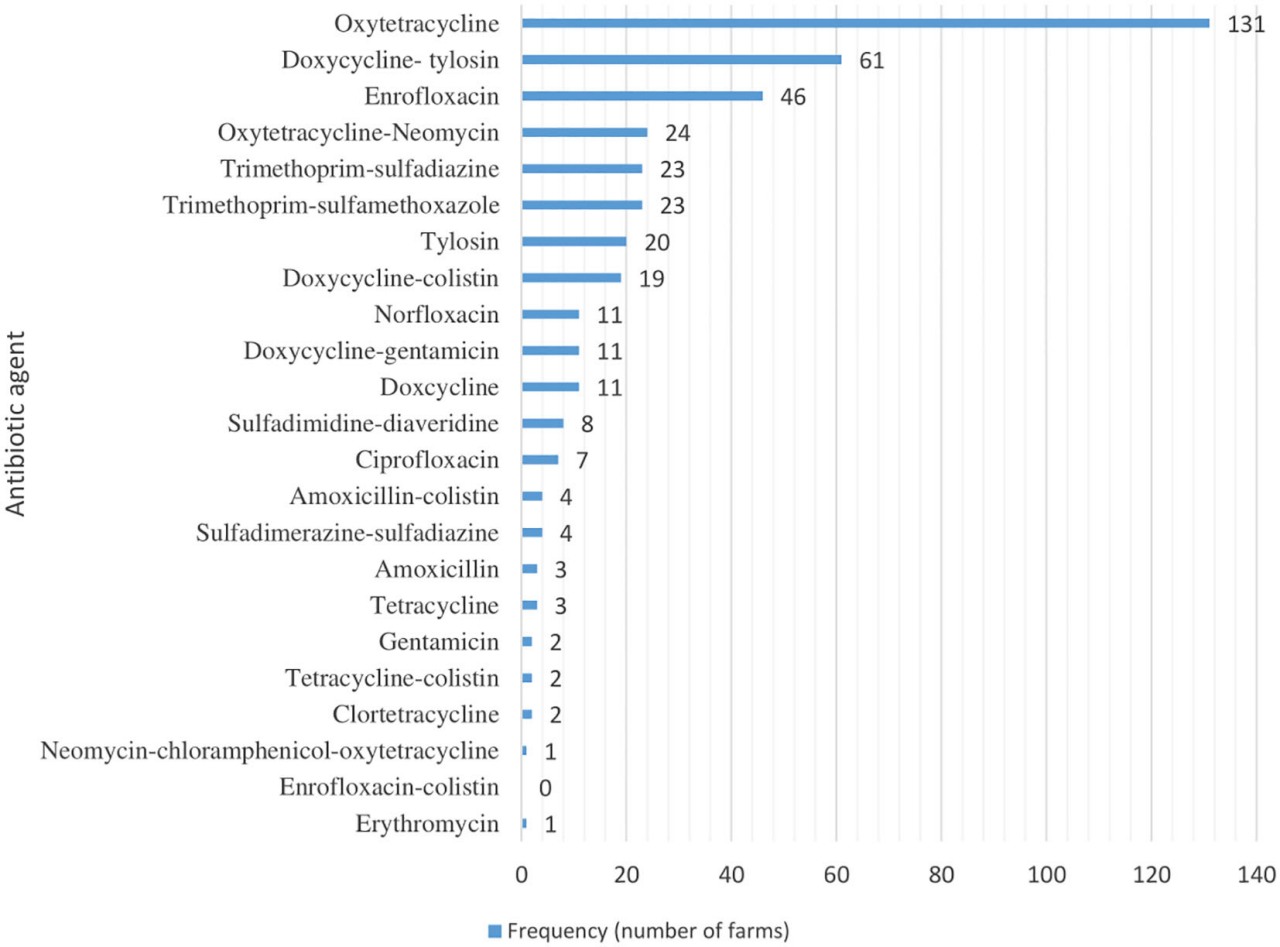
Distribution of antibiotic agents used in the selected farms.

A multistage sampling scheme was used to identify farms for the survey whereby the number of farms were randomly selected proportional to the targeted sample size for each study site from the sampling frame of 398 farms. In this case, an initial list of 207 farms was generated accounting for 10% nonresponse. The Livestock extension officers were used to give direction in reaching the farms for questionnaire administration. Eventually, a total of 205 farms were surveyed. The surveyed farms were then classified into small-scale (farms with 200 - 500 birds), medium-scale (farms with 501-2000 birds) and large-scale (farms with more than 2000 birds) as previously described and recommended (FAO, 2019; United Republic of Tanzania, 2019). During data collection, different brands of antibiotics were reported by the respondents to be used in the farms. The brands were expanded into essential antibiotic agents (**Supplementary Table S1**) and categorized into antibiotic classes (**Figure 1**) as previously described (WOAH, 2024).

Twenty-two statements were used to assess the practices that could influence antimicrobial stewardship (**Supplementary Table S3**). The items were combined to generate a composite score to determine the overall practice. The most appropriate responses were rated 1, incorrect responses or nonresponses rated 0. The total score for each respondent was obtained by summating the raw scores of each statement. The scores ranged from 5 to 17. Eight statements were used to assess the knowledge that could influence antimicrobial stewardship (**Supplementary Table S4**). The items were combined to generate a composite score to determine the overall knowledge. Each correct response was rated 1, incorrect response or nonresponse rated 0. The total score for each respondent was obtained by summing the raw scores of each statement. To assess the attitude that could influence antimicrobial use, four statements were used (**Supplementary Table S5**). The responses were measured on a two-point Likert scale (1 = disagree, 2 = agree). Negative statements were reverse-coded. A mean score for each respondent was calculated from all the four item responses to obtain a summated (Likert scale) score of the attitude. The mean score ranged from 1.00 to 2.00. The total mean scores were then presented in percentages.

### 2.5 Data analysis

Data were cleaned, coded and analysed using Microsoft Office Excel 2016 and IBM SPSS Statistics for Windows, Version 25.0. Armonk, NY: IBM Corp. Descriptive statistics (percentages, frequencies, medians and means) were used to describe the proportions of farm characteristics, respondent sociodemographic characteristics, KAP and antimicrobial use. For analysis purpose, participants were categorized according to the 2022 National Census in Tanzania (NBS, 2022) into young adults (18 – 35 years old), middle aged adults (36 – 59 years old) and older adults (60 years old and above). Chi square test was used to test the association between categorical variables. Non-parametric Kruskal-Wallis H test, with significance values adjusted by the Bonnferroni correction for multiple tests and Man-Whitney test were used to test the differences in the KAP scores between groups in variables with more than two categories and those with two categories respectively. Pearson correlation test was conducted to evaluate the significance, strength and direction of relationship between each pair of KAP scores. Differences among farm/sociodemographic characteristics for KAP scores were analysed using one-way analysis of variance (ANOVA) with Tukey HSD method for *post hoc* comparison. A multiple linear regression model was used to test the association between KAP scores (dependent variables) and farm/sociodemographic characteristics (independent variables). Frequency of antibiotic treatment regimens (dependent variable) was considered as count variable, and to account for the overdispersion, as the variance exceeded the mean, a negative binomial regression (NBR) model (Coxe et al., 2009) was used to test its relationship with the farm/sociodemographic characteristics (independent variables).

## 3 Results

### 3.1 Sociodemographic and farming characteristics of the respondents

**Table 1** shows the sociodemographic and farming characteristics of the respondents. The majority (72.7%) of the respondents were from Dar es Salaam and mainly (82.9%) comprised of farm owners. More than a half of the participants were female (55.1%, n = 113). The age of the participants ranged from 18 to 74 years with an overall mean age of 47 years (STD:13). Commercial layer farm size ranged from 200 to 143,000 layer chickens with a median of 500 (IQR: 275 - 1000). More than a half (50.2%) of the farms were small scale and majority (61.0%) had laying chickens only.

**Table 1:** Sociodemographic and farming characteristics of the participants (N = 205).

Variable (Characteristic)	Category	Number (%)
Study site	Dar es Salaam	149 (72.7)
	Morogoro	35 (17.1)
	Unguja	21 (10.2)
Sex	Male	92 (44.9)
	Female	113 (55.1)
Position in the farm	Owner	170 (82.9)
	Manager	21 (10.2)
	Supervisor	14 (6.8)
Age group (yrs)	Young adults (18–35)	45 (22.0)
	Middle-aged adults (36–59)	120 (58.5)
	Old adults (60–74)	40 (19.5)
Education level	Primary	62 (30.2)
	Secondary	87 (42.4)
	Tertiary	56 (27.3)
Experience in keeping layers	≤ 2 years	50 (24.4)
	> 2 years	155 (75.6)
Scale of production	Small scale (200-500 birds)	103 (50.2)
	Medium scale (501-2000 birds)	81 (39.5)
	Large scale (>2000 birds)	21 (10.2)
Chicken composition (age groups) per farm	Chicks only	10 (4.9)
	Pullets only	14 (6.8)
	Laying chicken only	125 (61.0)
	Laying + chicks	25 (12.2)
	Laying + pullets	21 (10.2)
	Laying + pullets + chicks	10 (4.9)

### 3.2 Prevalence and characteristics of the antimicrobial agents used in layer chickens

The majority (97.1%, n = 199) of the respondents reported using antibiotic agents during the period of three months preceding the study visit.

#### 3.2.1 Essential antibiotic agents used

**Figure 2:**
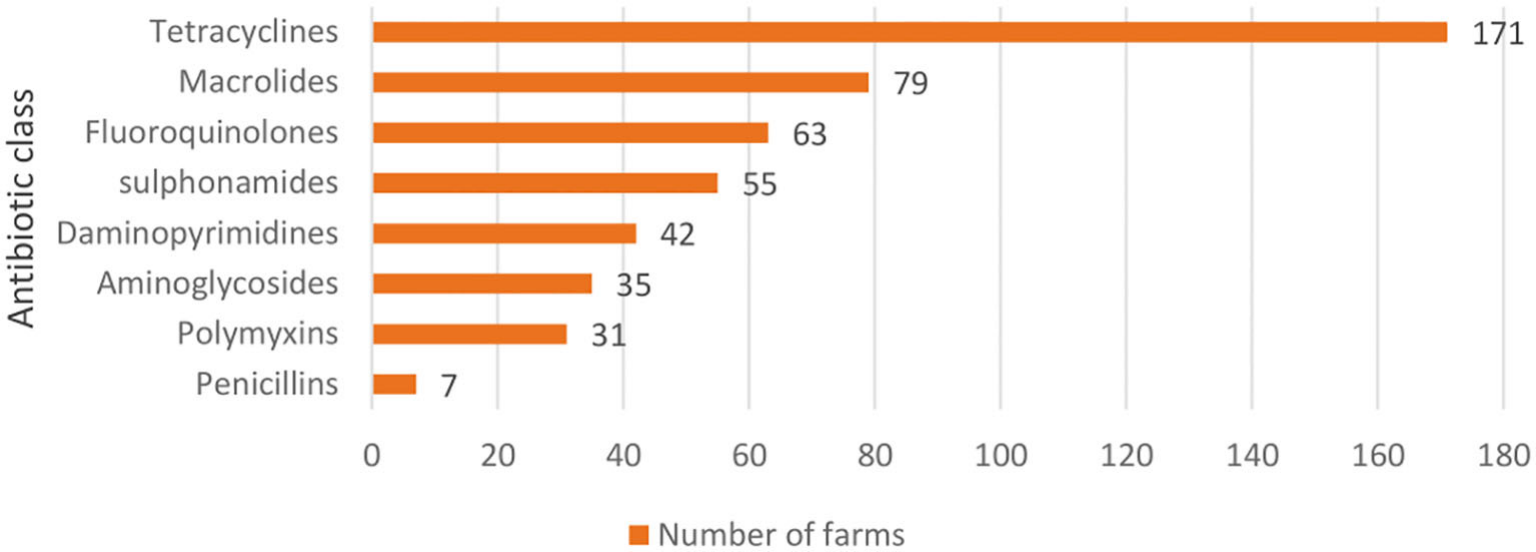
Classes of antibiotic agents used in the study farms.

A total of 23 antibiotics (alone or in combination) were used in the three months prior to the study (**Figure 2**). Oxytetracycline was the most commonly used antibiotic (63%, n= 131), followed by doxycline-tylosin combination (29.8%, n = 61) and enrofloxacin (22.4%, 46). The frequency of antibiotic administration (treatment regimens) varied considerably among farms, with an overall median of 4 treatments (IQR: 2 - 7).

#### 3.2.2 Antibiotic classes

The classes of antibiotic agents used (alone or in combination) in the study farms are shown in **Figure 1**. Tetracyclines (n = 171) were reported to be used by most of the respondents in their farms. The use of diaminopyrimidines, polymixins and penicillins was not reported in the farms which had only chicks during the study visit, however, these farms reported higher number of treatment regimens with tetracycline class of antibiotics and was followed by those with pullets only (**Supplementary Table S2**).

#### 3.2.3 Purpose of antibiotic use

**Figure 3:**
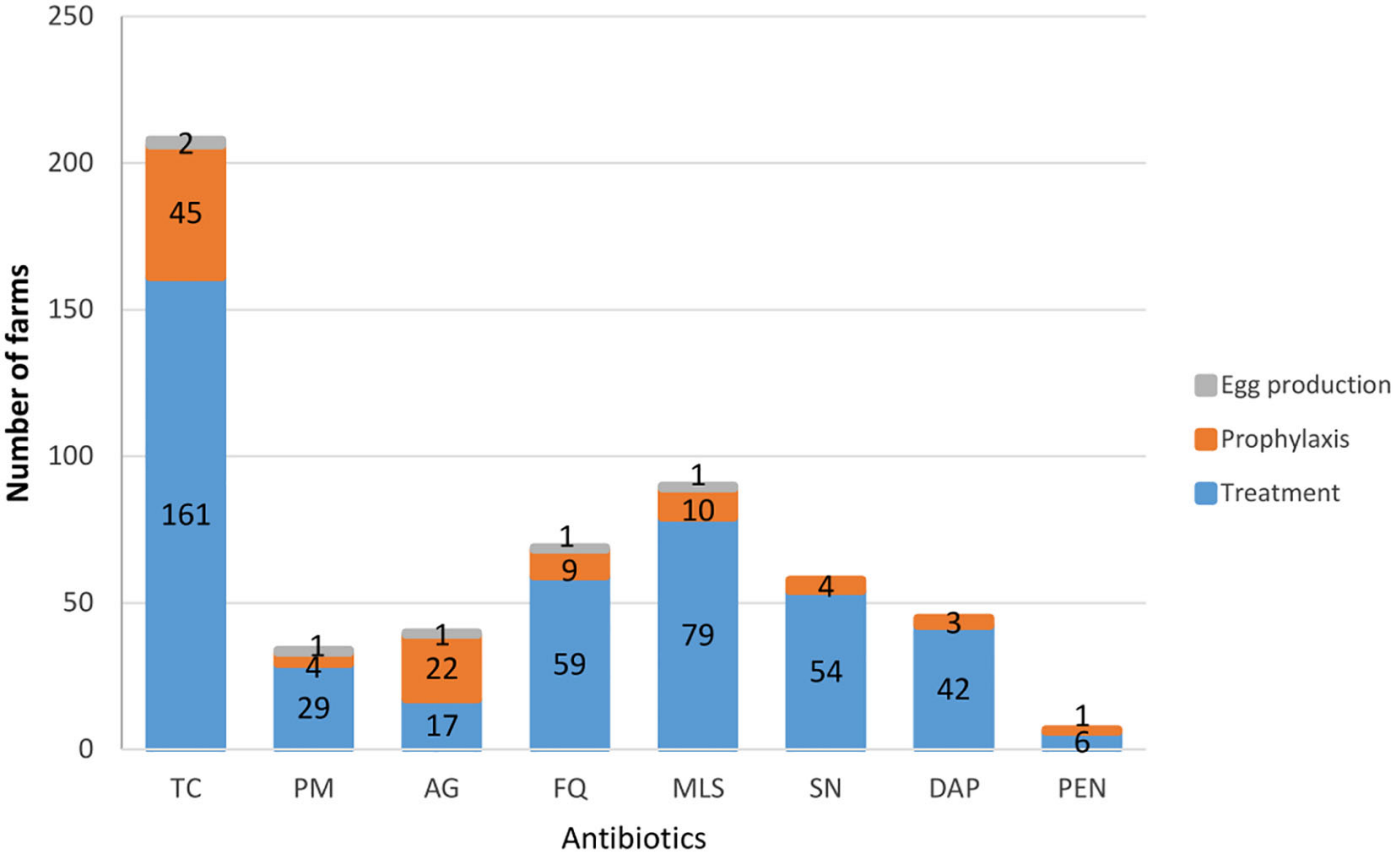
Purpose of antibiotic agents use in the farms TC, Tetracyclines; PM, Polymixins; AG, Aminoglycosides; FQ, Fluoroquinolones; MLS, Macrolides; SN, Sulphonamides; DAP, Diaminopyrimidenes; PEN, Penicillins.

Antibiotics were used at different levels for treatment, prophylaxis and/or increasing egg productivity (**Figure 3**). Of the total 40 farms that used aminoglycoside-containing agents, 22 (55.0%) used them for prevention, 17 (42.5%) treatment and 1 (2.5%) for improving egg production.

### 3.3 Practices related to the use of antibiotics

About half (55.1%, n = 113) of the respondents reported to seek advice from an animal health professional (AHP) when their birds were sick, and further the majority of respondents (66.3%, n = 136) consulted experts for the type of antibiotic to use in their farms. It was reported that antibiotics were mostly (99.0%, n = 203) obtained from veterinary drug stores and were administered by farm owners (68.5%, n = 126), farm managers (10.3%, n = 19), farm supervisors (6.0%, n = 11), animal health professionals (6.0%, n = 11) or other farm workers (9.2%, n = 17).

When sick chickens failed to respond to treatment, nearly half (44.0%, n = 81) of the respondents reported to change to another type of antibiotic while only 29.9% of respondents (n=55) would consult an AHP for second opinion. The respondents reported that expired antibiotics were mostly thrown away (37.7%, n = 77), buried (26.3%, n = 54) or burnt (19.0%, n = 39). Further, a large proportion (95.6%, n = 196) of the respondents did not abide to withdrawal period and they kept on selling eggs from chickens under antibiotic treatments.

### 3.4 Knowledge on antibiotic use

The majority of respondents (86.3%), n = 177 knew what antibiotics were and their uses (**Supplementary Table S4**). Half of the respondents (50.2%, n = 103) reported to know the withdrawal time and 86 (41.9%) of all respondents could correctly define the withdrawal period. The majority (92.2%, n = 189) of respondents reported that they can identify the expired antibiotics by checking on the printed expiry date on the label.

### 3.5 Attitude towards antibiotic use

The majority (67.3%, n = 138) agreed with the statement “It is not reasonable to throw away eggs from chicken during or directly after treatment with antibiotics” (**Supplementary Table S5**). More than a half of the respondents (55.1%, n = 113) agreed with the statement “Drugs are expensive so you should use them for as long as you can”. Only a small proportion of respondents (43.4%, n = 89) disagreed with the statement that “Antibiotics should be used only for the purpose of curing diseases”.

### 3.6 Relationship between essential antibiotic use, KAP and sociodemographic/farm characteristics

Kruskal-Wallis H test adjusted by Bonferroni correction indicated that among the essential antibiotics used in the farms, medium-scale farms used a significantly higher number of treatment regimens with tetracyclines than small-scale farms (*p* = .011). Similarly, the number of treatment regimens with polymixins was significantly higher in medium-scale farms than in the small-scale (*p* = .018). Large-scale farms used a higher number of treatment regimens for diaminopyrimidines than the medium-scale (*p* = .029) and small-scale (*p* = .037) farms. Among the essential antibiotics used in the farms, the number of treatment regimens with oxytetracycline was significantly higher in the medium-scale farms than in the small-scale farms (*p* = .002). Similarly, medium-scale farms had higher number of treatment regimens with colistin-docycycline combination than small-scale farms (*p* = .035). Further the knowledge was found to be positively correlated with practice (*r* = 0.362, P <.001) and practice was positively correlated with attitude (*r* = 0.221, *p* = .001).

**Table 2** shows One-way ANOVA test results on the relationship between KAP and farm/sociodemographic characteristics. Tukey’s multiple comparisons *post hoc* test revealed that respondents from Morogoro scored higher on knowledge compared to those from Unguja (*p* = .037). Females (*p* = .013) and older adults (*p* = .013) scored significantly higher than male and middle-aged adults respectively on attitude towards antibiotic use. Furthermore, respondents from Dar es Salaam (*p* = .013) and Morogoro (*p* = .024) reported significantly higher on the attitudes toward the uses of antibiotics in chicken. Also, respondents from Dar es Salaam (*p* <.001) and Morogoro (*p* = .002) reported significantly higher than the respondents from Unguja on the practices related to the use antibiotics.

**Table 2:** Distribution of knowledge, attitude and practice among sociodemographic/farm characteristics.

Variable (Characteristic)	Category	Knowledge			Practice			Attitude		
		% Mean	SD	*p-*value*	% Mean	SD	*p-*value*	% Mean	SD	*p-*value*
Geographical location	Unguja	47.0	24.0	**0.046**	40.5	13.4	**< 0.001**	62.5	8.8	**0.013**
	Morogoro	62.0	20.8		50.8	10.9		70.4	13.9	
	Dar es Salaam	55.1	22.1		52.4	10.3		69.7	10.2	
Age group	Young adult	56.8	22.3	0.661	52.4	11.0	0.586	70.6	8.9	**0.007**
	Middle-aged adult	54.3	21.4		50.4	11.7		67.2	10.6	
	Older adult	57.5	25.2		50.6	10.5		73.1	12.8	
Sex	Male	53.0	23.8	0.145	49.8	12.5	0.215	67.0	11.5	0.13
	Female	57.5	20.9		51.8	10.1		70.8	10.2	
Education level	Primary	53.9	22.0	0.802	51.2	10.2	0.956	71.0	10.3	0.131
	Secondary	56.3	22.8		50.7	12.2		67.4	10.2	
	Tertiary	55.9	22.2		50.7	11.1		70.0	12.6	
Production scale	Small-Scale	53.7	16.8	0.925	53.5	10.2	0.139	67.9	10.9	0.84
	Medium-scale	55.9	22.7		49.0	10.1		69.4	9.7	
	Large-scale	55.5	23.1		51.8	12.2		69.1	12.0	
Experience	≤ 2 years	56.2	22.2	0.387	50.8	11.4	0.925	68.6	11.4	0.249
	> 2 years	53.1	22.8		51.0	11.1		70.7	9.4	

*One-way ANOVA.Bold font indicates statistically significant difference between group means.

### 3.7 Factors influencing the frequency of antimicrobial use in the farms

**Table 3** shows the negative binomial regression analysis conducted to assess the influence of farm and farmer characteristics on antibiotic treatment frequency. The model showed significant fit (χ2 = 24.902, *p* = .009). The model’s appropriateness was further supported by a reduction in Akaike’s Information Criterion (AIC) from 1463.975 (Poisson, dispersion = 5.009) to 1122.229 (NBR, dispersion = 1.254), indicating successful handling of over dispersion in the data.

**Table 3:** Negative binomial regression predicting the likelihood of the frequency of antibiotic use in commercial chicken farms.

Parameter	Category	B	Std. Error	*p* - value	Exp(B)	95% CI	
						Lower	Upper
Geographical location	(Intercept)	0.993	0.2726	**0.000**	2.699	1.582	4.605
	Dar es Salaam	0.365	0.2183	0.094	1.441	0.939	2.210
	Morogoro	0.647	0.2484	**0.009**	1.910	1.174	3.108
	Unguja (reference)						
Gender	Male	-0.056	0.1302	0.666	0.945	0.732	1.220
	Female (reference)						
Age	Young adult	0.540	0.1976	**0.006**	1.717	1.165	2.528
	Middle-aged adults	0.205	0.1676	0.222	1.227	0.884	1.704
	Older adults (reference)						
Education level	Primary	0.029	0.1650	0.863	1.029	0.745	1.422
	Secondary	-0.012	0.1550	0.939	0.988	0.729	1.339
	Tertiary (reference)						
Experience	≤ 2 years	-0.178	0.1515	0.241	0.837	0.622	1.127
	> 2 years (reference)						
Household size	≤4 persons	-0.237	0.1433	0.098	0.789	0.596	1.045
	≥5 persons (reference)						
Scale of production	Small-scale	0.258	0.2184	0.238	1.294	0.843	1.985
	Medium-scale	0.378	0.1318	0.004	1.459	1.127	1.889
	Large-scale (reference)						

Bold font indicates significant association between variables.

Analysis of individual predictors revealed significant associations between treatment frequency and three key variables: farmer age, farm scale, and geographical location. Young adult farmers showed significantly higher treatment frequencies compared to older adults (β = 1.717, *p* = .006), with an expected increase of 457% in treatment frequency (exp(β) = 5.57), holding other variables constant. Medium-scale farms demonstrated higher treatment frequencies compared to large-scale farms (β = 1.459, *p* = .004), with an expected increase of 330% in treatment frequency (exp(β) = 4.30). Farms located in Morogoro showed higher treatment frequencies compared to those in Unguja (β = 1.910, *p* = .009), with an expected increase of 575% in treatment frequency (exp(β) = 6.75).

### 3.8 Factors influencing KAP in commercial layer farms

**Table 4** shows results of a multiple linear regression conducted to analyse the effect of scale of production, sex, age, experience, education and position of the person who plays a primary role in the management of chickens on KAP. Farms located in Morogoro were associated with 0.244 scores increase in knowledge (β = 0.244, t = 2.228, *p* = .027) compared to those in Unguja. Attitude scores were 0.224 higher in farmers located in Dar es Salaam (β = 0.224, t = 2.126, *p* = .035) than those in Unguja. Farmers with primary education demonstrated 0.161 scores higher in attitude than those with secondary level of education (β = 0.161, t = 2.137, *p* = .034). Further, older adult farmers showed a 0.206 units higher altitude score as compared to middle-aged adults ((β = .206, t = 2.728, *p* = .007). Practices scores were 0.495 higher in farmers located in Dar es Salaam (β = 0.495, t = 4.725, *p* < 0.001) and 0.380 higher in farmers located in Morogoro (β = 0.380, t = 3.633, p < 0.001) than those located in Unguja. On the contrary, small-scale farms demonstrated 0.152 lower scores on practices compared with large-scale farms (β = -0.152, t = -2.133, p = 0.034). These results implies that farm scale, age of the farmer, education of the farmer and geographical location are significant predictors of KAP.

**Table 4:** Multiple linear regression showing the effects of farm/sociodemographic characteristics on KAP scores.

Parameter	Category	Knowledge			Attitude			Practices		
		β	*t*	*P* value	β	*t*	*p* value	β	*t*	*p* value
Geographical location	(Intercept)		6.977	0.000		20.299	0.000		12.235	0.000
	Unguja (reference)									
	Morogoro	0.244	2.228	**0.027**	0.169	1.607	0.110	0.380	3.633	**0.000**
	Dar es Salaam	0.156	1.425	0.156	0.224	2.126	**0.035**	0.495	4.725	**0.000**
Sex	Male (reference)									
	Female	0.061	0.810	0.419	0.108	1.503	0.135	0.0003	0.039	0.969
Age	Middle-aged adults (reference)									
	Young adults	0.075	0.943	0.347	0.094	1.235	0.218	0.056	0.742	0.459
	Older adults	0.015	0.191	0.849	0.206	2.728	**0.007**	0.019	0.256	0.799
Education level	Secondary (reference)									
	Primary	-0.065	-0.821	0.413	0.161	2.137	**0.034**	-0.031	-0.411	0.682
	Tertiary	-0.022	-0.27	0.788	0.086	1.121	0.264	-0.047	-0.613	0.541
Experience	≤ 2 years (reference)									
	> 2 years	-0.056	-0.734	0.464	0.064	0.873	0.383	-0.019	-0.257	0.797
Household size	> 4 persons (reference)									
	≤ 4 persons	0.089	1.176	0.241	-0.059	-0.810	0.419	0.043	0.593	0.554
Scale of production	Small-scale (reference)									
	Medium-scale	-0.011	-0.152	0.879	0.003	0.042	0.966	-0.152	-2.133	**0.034**
	Large-scale	-0.047	-0.596	0.552	-0.039	-0.505	0.614	0.030	0.401	0.689

Bold font indicates statistically significant association between respective variables.

## 4 Discussion

The purpose of the present study was to explore the factors that could influence frequency, knowledge, attitude and practices of antibiotic use on commercial layer farms. To our knowledge, this is the first study to address factors influencing the frequency of antibiotic usage in commercial layers of Tanzania. Several antibiotics were reported by the majority of the respondents to be frequently used in layers. The study has revealed that farmers’ geographical location, level of production and age may be influencing factors that affect how antibiotics are used at a farm level. A few other studies have reported the frequency of antibiotic use in animals elsewhere including Kenya and Uganda (Kisoo et al., 2023; Kibooga et al., 2024). A recent study in Kenya (Rware et al., 2024) reported a frequency of more than 20 times administration of antibiotics in chickens per month. Frequent administration of antibiotics in layer chickens can result into production of eggs with antimicrobial residues potentiating the spread of antibiotic resistance to humans (Rahman et al., 2022; Lima et al., 2023).

In the present study the frequency of antibiotic use varied between locations which may be associated with the variation in the incidence of infections (Ngongolo, 2024), limited availability of veterinary services or easy access to antibiotics (Kibooga et al., 2024) and the influence of information spill over among farmers clustering in close proximity (Omondi, 2022). The high use of antibiotics in Morogoro may be linked to high disease occurrences in the area. The present study has revealed that medium scale farmer use more antibiotics than large-scale farms. The observed difference could be attributed to the differences in the disease management strategies utilized between the farms. Farmers who keep large number of poultry tend to have higher investment in disease prevention strategies eventually affecting the frequency of antibiotic use in the farms (Adeyonu et al., 2021).

The study further revealed that age has an influence on the frequency of antibiotic use regimen whereby young adult respondents use antibiotics more frequently in their layer chickens than older adults. A previous study in Dar es Salaam indicated that young farmers were characterised with more imprudent use of antibiotics than adult farmers (Azabo et al., 2022). High use of antibiotics in farms supervised by young adults may be linked with the level of risk perception and knowledge of antibiotics and subsequently the farming management practiced by the young adults (Naghavi et al., 2024). Studies show that age of the farmer plays an important role in the adoption of the new farm management practices where by young adults embrace new technologies showing greater willingness to experiment with new practices (Brown et al., 2019; Udoh et al., 2024). In this context, the acceptance levels of novel or new information about antibiotic use may be higher among young adult farmers hence accelerating the frequency of antibiotic use in their farms.

The majority of the respondents reported to use antibiotics on layers for treatment, followed by prophylactic purposes. Twenty-three antibiotics (either alone or in combination) were reported to be used in the study area, including colistin and fluoroquinolones, which are considered as “critically important” both for human and animal health (WHO, 2019; WOAH, 2024). It is recommended that fluoroquinolones and colistin should neither be used prophylactically nor as first line treatments, rather their use should be only justified after bacteriological testing and it’s use is prohibited for animal usage in many counties. Neomycin, which is among the “veterinary critically important antimicrobial agents”, was also used more frequently in combination with oxytetracycline for prophylactic purposes. Prophylactic use of antibiotics in poultry as an alternative disease prevention strategy has been reported in various countries (Imam et al., 2020). Irrational use of these antibiotics in commercial layers may promote the development of antibiotic resistance posing great concern both in humans and in animals. It is therefore critically important that proper diagnosis be made before any use of the antibiotics to avoid the development of resistance.

The high use of oxytetracyclines, as observed in this study, has been reported previously in Tanzania (Mubito et al., 2014b; Chota et al., 2021; Azabo et al., 2022) and other developing countries (Mela et al., 2021; Mutua et al., 2023). The wide use of oxytetracycline has been linked to its broad-spectrum nature of action, lower price and availability (Ferdous et al., 2020). Excessive use of oxytetracycline has been linked with the development of resistance genes in pathogenic microorganisms (Sudha et al., 2022), therefore ways identifying ways to reduce its use are necessary to ensure sustainable use of oxytetracycline. Studies show that many bacterial pathogens isolated from animals have developed high resistance against tetracyclines and this warrants for a review of its use (Nonga and Muhairwa, 2010; Mayenga et al., 2020; Mdegela et al., 2021; Sangeda et al., 2021).

Failure to observe antibiotic withdrawal periods in livestock poses significant public health risks through antibiotic residues in animal products entering the human food chain. In our study, most farmers did not respect the required withdrawal periods for antibiotics in their birds. This non-adherence to withdrawal periods in poultry aligns with findings from other developing countries (Guetiya Wadoum et al., 2016; Al-Mustapha et al., 2020; Habiba et al., 2023). Despite awareness of withdrawal period requirements, farmers reported that potential financial losses associated with egg disposal prevented compliance. This practice would lead to antibiotic-containing eggs being released into the market, consequently contributing to AMR development. Indeed, some studies on antibiotic residues in eggs have reported high prevalence of antibiotic residues in eggs (Nonga et al., 2009; Mubito et al., 2014a; Mosha, 2017). With this situation, the public is at danger of persistently eating eggs with such levels of residues with the outcome of high AMR and many other health effects associated with antibiotics in humans. Targeted market creation to ensure competitive pricing of antibiotic-free eggs may incentivize farmers to observe withdrawal periods and avoid unnecessary use of antibiotics; however, this strategy must be coupled with appropriate measures for controlling disease incidence in the farms to minimize the need for frequent use of antibiotics.

The multiple regression analysis revealed that knowledge on antibiotic use is influenced by the location of the farmer. The variation may be influenced by the differences in the availability of animal health services across geographical locations (Tufa et al., 2023). A similar study in Tanzania (Sindato et al., 2020) showed differences in the attitude between participants from Ilala, Kilosa and Kibaha. Similar results have been reported reported from other developing countries including Uganda, Kenya and Peru (Benavides et al., 2021; Kahunde et al., 2023; Rware et al., 2024) suggesting for a need to improve animal health services including access to laboratory for proper diagnosis to ensure appropriate antibiotic choice against infections.

The present study has also demonstrated that the attitude of the participants on the use of antibiotics is influenced by education level, age and location of the farmer. Specifically, farmers with primary level of education and older adults had a more favourable attitude on proper use of antibiotics than those with secondary level of education. Farmers with lower education are likely to have developed a tendency of higher reliance to the instructions provided by animal health professionals on disease management (Tufa et al., 2023). Similarly, older adult farmers are likely to have developed a better behaviour on proper use of antibiotics (Hassan et al., 2021). This calls for training programmes tailored to education level and age groups to improve antibiotic stewardship among layer farmers.

Overall, imprudent use of veterinary antibiotics has been frequently reported in developing countries raising various health concerns including AMR (Liyanage and Pathmalal, 2017; Coyne et al., 2019; Ferdous et al., 2019; Emes et al., 2023). It is imperative that developing countries should prioritize interventions aiming at adoption of infection prevention and control (IPC) measures such as biosecurity and vaccination among farmers to improve AMR stewardship. Furthermore, AMR mitigation policies in low-resource settings should be grounded on socio-economic issues aiming at improving knowledge, attitude and practices related to antimicrobial use among farmers.

### 4.1 Study limitations

The study is limited to three regions in Tanzania (Unguja, Morogoro, and Dar es Salaam), which may not fully represent the diversity of farming practices and challenges across the country. Extending the study to other regions in the country would provide a more comprehensive understanding of antibiotic use practices, potentially leading to more robust and generalizable insights. Additionally, the cross-sectional nature of the study limits the ability to establish causal relationships between the identified factors and antibiotic use practices. Longitudinal studies would provide stronger evidence for causality. Furthermore, the reliance on self-reported data from farmers introduces the potential for recall bias, especially regarding antibiotic use practices and frequencies. Although the study attempted to mitigate this by providing pictures of drugs, the accuracy of responses remains a concern. This could be mitigated by employing prospective study designs that allow collection of data as event occur, minimizing time lapses between events and data collection. However, these findings provide baseline evidence for designing interventions and policies regarding antibiotic use.

## 5 Conclusion

Knowledge, attitude and practice about the usage of veterinary antibiotics is essential in articulating appropriate interventions for mitigation of antibiotic resistance. Our study has identified the factors associated with the frequency of antibiotic administration and KAP on the commercial layer farms. The frequency, knowledge, attitude, practices related to the use of antibiotics were affected by scale of production, location, age, and education. Policy makers should consider tailored interventions to improve antibiotic stewardship at the farm level. Further research is needed to identify factors other than farm/social demographic characteristics such as economic influence and policy changes.

## Data Availability

The original contributions presented in the study are included in the article/**Supplementary Material**. Further inquiries can be directed to the corresponding author.
