# Supplementary material for: Factors influencing the frequency, knowledge, attitudes and practices of antibiotic use in commercial layer chicken farms, Tanzania
Source: Front Antibiot. 2025 Apr 28;4:1571096. doi: 10.3389/frabi.2025.1571096 (PMC12066604; doi:10.3389/frabi.2025.1571096)
Supplement: Supplementary file 1 [file Table1.doc]

| **Supplementary Table 1.** Essential antimicrobials used in the study farms | | | |
| --- | --- | --- | --- |
| **Antimicrobial** | **N = 199**  **n (%)** | **Median (IQR)** | **Range*** |
| **Antibiotics** |  |  |  |
| Oxytetracycline | 131 (65.8) | 2 (1 - 4) | 1 - 15 |
| Tetracycline | 3 (1.5) | 2 | 1 - 3 |
| Doxcycline | 11 (5.5) | 3 (2 – 10) | 1 - 15 |
| Clotetracycline | 2 (1.5) | 4 | 1 - 7 |
| Oxytetracycline-Neomycin | 24 (12.1) | 1 (1 – 4) | 1 - 8 |
| Tetracycline-colistin | 2 (1.5) | 1 | 1 - 2 |
| Doxycycline- tylosin | 61 (30.7) | 2 (1 – 3) | 1 - 10 |
| Doxycycline-colistin | 19 (12.3) | 1 (1 – 3) | 1 - 7 |
| Doxycycline-genta | 11 (5.5) | 1 (1 – 3) | 1 - 11 |
| Tylosin | 20 (10.1) | 1 (1 – 3) | 1 - 8 |
| Ciprofloxacin | 7 (2.5) | 1 (1 - 3) | 1 - 6 |
| Enrofloxacin | 46 (23.1) | 2 (1 -3) | 1 - 7 |
| Norfloxacin | 11 (5.5) | 3 (1 – 4) | 1 – 10 |
| Gentamicin | 2 (1.5) | 3 | 3 - 5 |
| Trimethoprim-sulfamethoxazole | 23 (11.6) | 2 (1 – 5) | 1 - 13 |
| Trimethoprim-sulfadiazine | 23 (11.6) | 1 (1 – 2) | 1 - 10 |
| Sulfadimidine-diaveridine | 8 (4.0) | 2 (1 – 3) | 1 - 4 |
| Sulfadimerazine-sulfadiazine | 4 (2.0) | 1 (1 – 3) | 1 - 3 |
| Amoxycillin | 3 (1.5) | 1 | 1 |
| Amoxycillin-colistin | 4 (2.0) | 1 (1-2) | 1 - 2 |
| Erythromycin | 1 (0.5) | 4 | 4 |
| Enrofloxacin-colistin | 1 (0.5) | 1 | 1 |
| Neomycin-cholompenicol-ocytetracycline | 1 (0.5) | 2 | 2 |
| **Antiparasites** |  |  |  |
| Amprolium | 54 (27.1) | 2 (1 – 3) | 1 - 7 |
| Toltrazuril | 7 (3.5) | 1 (1 – 2) | 1 - 9 |
| Ivermectin | 5 (2.5) | 1 (1 – 2) | 1 - 2 |
| Piperazine | 10 (5.0) | 1 (1 – 3) | 1 – 4 |
| Levamisole | 13 (6.5) | 1 (1 – 3) | 1 - 5 |

*minimum and maximum frequency of treatment regimens

| **Supplementary Table 2**. Distribution of treatment regimens with each of antibiotic classes among farms by chicken age group composition | | | | | | | |
| --- | --- | --- | --- | --- | --- | --- | --- |
|  | **Laying**  n (mean rank*) | **Pullets**  n (mean rank*) | **Chicks**  n (mean rank*) | **Laying+Pullets**  n (mean rank*) | **Laying+Chicks**  n (mean rank*) | **Laying+Pullets+Chicks**  n (mean rank*) | *P* value |
| Tetracyclines | 104 (84.7) | 12 (100.3) | 10 (107.6) | 15 (70.9) | 21 (84.0) | 9 (87.9) | .476 |
| Macrolides | 46 (42.3) | 9 (32.9) | 4 (39.0) | 4 (36.3) | 12 (35.1) | 4 (48.5) | .734 |
| Fluoroquinolones | 38 (32.2) | 6 (34.3) | 3 (32.2) | 6 (35.8) | 8 (28.3) | 2 (24.3) | .949 |
| Sulphonamides | 33 (27.2) | 5 (38.9) | 2 (26.8) | 3 (13.0) | 9 (31.5) | 3 (24.2) | .277 |
| Diaminopyrimidines | 24 (21.4) | 5 (24.9) |  | 2 (9.5) | 9 (22.4) | 2 (22.0) | .628 |
| Aminoglycosides | 24 (17.6) | 2 (17.5) | 1 (33.5) | 3 (11.0) | 4 (20.3) | 1 (24.0) | .363 |
| Polymixins | 23 (17.9) | 1 (21.0) |  | 3 (9.0) | 4 (9.0) |  | .084 |
| Penicillins | 5 (4.6) |  |  | 1 (2.5) | 1 (2.5) |  | .407 |

* Kruskal-Wallis H test

| **Supplimentary Table 3.** Practices related to antibiotic use | | |
| --- | --- | --- |
| **Statement** | **Correct response**  **n (%)** | **Incorrect response**  **n (%)** |
| Did you call for animal health expert in the last 12 months? (Yes) | 132 (64.4) | 73 (35.6) |
| Did you use laboratory services, for example, taking a sick or dead chicken to the laboratory in the last 12 months? (Yes) | 78 (38.0) | 127 (62.0) |
| What did you do with your layers when they fell sick? *(*Correct statement*: Consult animal health professional)* | 113 (55.1) | 92 (44.9) |
| How did you decide on the drug you used for treatment? *(Correct response: Advice from animal health professional/agrovet)* | 136 (66.3) | 69 (33.7) |
| Do your treatment choices differ for layers of different ages? (*Yes*) | 81 (39.5) | 124 (60.5) |
| Who administered the first most commonly used drug the last time you used it? *(Animal health service provider)* | 16 (7.8) | 189 (92.2) |
| Who administered the second most commonly used drug the last time you used it? (*Animal health service provider*) | 14 (6.8) | 191 (93.2) |
| Which chickens were given the first most commonly used drug the last time you used it? (*All chickens*) | 167 (81.5) | 38 (18.5) |
| Which chickens were given the second most commonly used drug the last time you used it? (*All chickens*) | 178 (86.8) | 27 (13.2) |
| Where did you obtain the first most commonly used drug from? (*From animal health service provider/agrovet*) | 203 (99.0) | 2 (1.0) |
| Where did you obtain the second most commonly used drug from? (*From animal health service provider/agrovet*) | 203 (99.0) | 2 (1.0) |
| How did you know how to use the first most commonly used drug? (*From animal health service provider/agrovet* | 181 (88.3) | 24 (11.7) |
| How did you know how to use the second most commonly used drug? (*From animal health service provider/agrovet* | 170 (82.9) | 35 (17.1) |
| Where specifically do you normally keep the first most commonly used drug (s) bought? (in a cabinet/cupboard/shelf/specific container) | 141 (68.8) | 64 (31.2) |
| Where specifically do you normally keep the second most commonly used drug (s) bought? (*in a cabinet/cupboard/shelf*) | 141 (68.8) | 64 (31.2) |
| Do you keep records of treatments of your chicken (layers)? (Yes) | 92 (44.9) | 113 (55.1) |
| What do you do with eggs collected from layers under and after a few days after treatment? (stop selling/human consumption) | 9 (4.4) | 196 (95.6) |
| Do you stop selling eggs when you use antibiotics? (*Yes*) | 17 (4.4) | 196 (95.6) |
| Do you stop consuming eggs when you use antibiotics? (*Yes*) | 32 (15.6) | 173 (84.4) |
| What do you do if sick layers under treatment do not improve?(Consult animal health professional) | 53 (25.9) | 152 (74.1) |
| What do you do if sick layers under treatment die a few days after treatment? (burry) | 125 (61.0) | 80 (39.0) |
| What do you do with expired drugs? (taking them back to the agrovet shop/municipal) | 13 (6.3) | 192 (93.7) |

| **Supplementary table 4.** Knowledge related to antibiotic use | | |
| --- | --- | --- |
| **Statement** | **Correct response**  **n (%)** | **Incorrect response**  **n (%)** |
| Do you know what antibiotics are? *(Yes)* | 177 (86.3) | 28 (13.7) |
| Do your treatment choices differ for layers of different ages? *(Yes)* | 81 (39.5) | 124 (60.5) |
| If YES: Why do your treatment choices differ? (correct statement: *Some drugs are not appropriate to certain chicken age*) | 31 (38.3) | 50 (61.7) |
| What are the reasons to stop using an antibiotic*? (End of treatment course)* | 129 (62.9) | 76 (37.1) |
| Do you know what withdrawal time is? (*Yes)* | 103 (50.2) | 99 (48.3) |
| How would you define withdrawal time? (n = 103)  *(Correct definition: time interval from administration of a antimicrobial agent to animal until consumption of animal product to assure that drug residues are below the maximum residue limit* | 86 (83.5) | 17 (16.5) |
| How do you identify expired antibiotics?*( correct response: through the printed expiry date)* | 189 (92.2) | 16 (7.8) |

| **Supplementary table 5.** Attitude related to antibiotic use | | |
| --- | --- | --- |
| **Statement** | **Agree** | **Disagree** |
| Drugs are expensive so you should use them for as long as you can | 113 (55.1) | 92 (44.9) |
| It is not reasonable to throw away eggs from chicken during or directly after treatment with antibiotics | 138 (67.3) | 67 (32.7) |
| Antibiotics should be used only for the purpose of curing diseases | 116 (56.6) | 89 (43.4) |
| Antibiotics may become less effective due to AMR | 38 (18.5) | 167 (81.5) |
